# 7-Tesla MRI Evaluation of the Knee, 25 Years after Cartilage Repair Surgery: The Influence of Intralesional Osteophytes on Biochemical Quality of Cartilage

**DOI:** 10.1177/19476035211060506

**Published:** 2021-11-26

**Authors:** M.P.F. Janssen, M.J.M. Peters, E.G.M. Steijvers-Peeters, P. Szomolanyi, E.M.C. Jutten, L.W. van Rhijn, L. Peterson, A. Lindahl, S. Trattnig, P.J. Emans

**Affiliations:** 1 Department of Orthopaedic Surgery, CAPHRI School for Public Health and Primary Care, Maastricht University Medical Center+, Maastricht, The Netherlands; 2 Scannexus Ultra High-Field MRI Center, Maastricht, The Netherlands; 3 High-Field MR Center, Department of Biomedical Imaging and Image-Guided Therapy, Medical University of Vienna, Vienna, Austria; 4 Department of Laboratory Medicine, Institute of Biomedicine, Sahlgrenska Academy, University of Gothenburg, Gothenburg, Sweden; 5 Sahlgrenska Academy, University of Gothenburg, Gothenburg, Sweden

**Keywords:** cartilage repair, knee, perichondrium, ACT/ACI, 7T MRI

## Abstract

**Objective:**

To evaluate the morphological and biochemical quality of cartilage transplants and surrounding articular cartilage of patients 25 years after perichondrium transplantation (PT) and autologous chondrocyte transplantation (ACT) as measured by ultra-high-field 7-Tesla (7T) magnetic resonance imaging (MRI) and to present these findings next to clinical outcome.

**Design:**

Seven PT patients and 5 ACT patients who underwent surgery on the femoral condyle between 1986 and 1996 were included. Patient-reported outcome measures (PROMs) were assessed by the clinical questionnaires: Knee injury and Osteoarthritis Outcome Score (KOOS), International Knee Documentation Committee (IKDC), and Visual Analogue Scale (VAS) for knee pain. The morphological (MOCART score) and biochemical quality (glycosaminoglycans [GAGs] content and collagen integrity) of cartilage transplants and surrounding articular cartilage were analyzed by 7T MRI. The results of the PT and ACT patients were compared. Finally, a detailed morphological analysis of the grafts alone was performed.

**Results:**

No statistically significant difference was found for the PROMs and MOCART scores of PT and ACT patients. Evaluation of the graft alone showed poor repair tissue quality and high prevalence of intralesional osteophyte formation in both the PT and ACT patients. Penetration of the graft surface by the intralesional osteophyte was related to biochemically damaged opposing tibial cartilage; GAG content was significantly lower in patients with an osteophyte penetrating the graft surface.

**Conclusions:**

Both PT and ACT patients have a high incidence of intralesional osteophyte formation 25 years after surgery. The resulting biochemical damage to the opposing tibial cartilage might be dependent on osteophyte morphology.

## Introduction

Knee injuries are very common and often seen in otherwise healthy, active patients.^1^ Several surgical treatments for focal cartilage defects have been developed aiming to prevent further deterioration of the knee joint, provide pain relief, and increase functional outcomes.^2^ Two of these techniques are perichondrium transplantation (PT) and autologous chondrocyte transplantation (ACT), which aimed at restoring the hyaline cartilage tissue using a perichondrium flap or cultured chondrocytes combined with a periosteum flap respectively.^3-7^

Short-term follow-up results of PT were reported by Homminga *et al.* and Bouwmeester *et al.* who concluded that the outcome of the surgery was poor.^5,7^ Long-term results of PT were described by Janssen *et al.*, who found that patient characteristics (i.e., time of symptoms prior to surgery, previous surgery in the index knee, and patient age) influence the outcome of PT at a follow-up of 22 years.^8^ The previous results of ACT were described by Peterson *et al.*, who found that after 10 to 20 years of follow-up, 92% of the patients were satisfied and would have the surgery again.^9^ Intralesional osteophytes occurred frequently after both PT and ACT.^10,11^ The cause of the frequent occurrence of intralesional osteophytes was not specifically investigated, but previous marrow stimulation techniques and the osteogenic potential of perichondrial and periosteal tissue are described to increase their occurrence.^12,13^ Increased calcification of cartilage repair tissue is known to impair the outcome of the surgery on a short-term follow-up.^14^ This impaired outcome is expected to persist at the long-term follow-up, but long-term results of cartilage repair surgery are scarce in literature. However, they are of great value to assess whether the initial goals of surgery were achieved.

Postoperative evaluation of cartilage repair tissue is important to assess the performance of cartilage repair procedures and to evaluate the different phases of repair, function, and degradation over time. Close insights in these phases will lead to better understanding of the process and improve cartilage repair strategies. Conventional modalities to follow patients after cartilage repair surgery include plain radiography and magnetic resonance imaging (MRI). Conventional radiography can sometimes visualize intralesional osteophytes and is helpful in grading the degree of late osteoarthritis (OA) as it visualizes joint space narrowing, osteophytes, sclerosis, and bony remodeling as a result of cartilage loss. MRI provides direct visualization of articular cartilage and surrounding soft-tissue structures, as well as bone marrow edema that can be involved in the OA disease process,^15^ and allows for a comprehensive evaluation of repair tissue from the articular joint surface to the bone-cartilage interface and the subchondral bone.

In 2017, the first 7 Tesla (7T) MR scanner (TERRA, Siemens Healthineers, Erlangen, Germany) was approved by the U.S. Food and Drug Administration (FDA) and Conformité Européenne (CE) certified in Europe, thus translating the so far experimental ultra-high-field MR (7 Tesla) into clinical routine examinations of the knee joint. With 7T MR, significantly higher, signal-to-noise ratios can be achieved compared to 3 Tesla, which provides higher spatial resolution in morphological imaging by a mean factor of 2.^16^ The higher signal-to-noise ratio allows depiction of small fissures and incomplete cartilage repair tissue integration^17^ and the detection of smaller physiological effects. On the downside, challenges of scanning at higher field strength include faster heating of tissue (specific absorption rate [SAR] limits), more intense metallic artifacts and more susceptibility artifacts at the transition between tissues with different densities caused by more field heterogeneities.^18^ The added value of 7T MRI lies within dedicated quantitative MR techniques that allow measurement of the biochemical properties of cartilage. Healthy cartilage is characterized by a high concentration of glycosaminoglycans (GAGs) and a well-organized collagen network. Both the GAG content and the organization of the collagen network are important indicators for repair tissue quality after treatment.^15^ GAGs carry protons that are in constant chemical exchange with surrounding bulk water protons. Using high-field MRI and a dedicated GAG Chemical Exchange Saturation Transfer (gagCEST) imaging sequence, these protons bound to GAG can be selectively labeled by saturation with a radiofrequency pulse. The label will then be transferred to the bulk water by chemical exchange which results in a reduction of the bulk water signal. This reduction in signal is a measure for the ratio of protons bound to GAG and the bulk water protons and is thereby an indirect measure for the GAG content.^19^ An advantage of gagCEST is that it can be performed without a contrast agent and using a regular proton coil, as opposed to dGEMRIC which requires a contrast agent and sodium imaging which requires a sodium coil to assess the GAG content. On the downside, the acquisition and postprocessing steps of gagCEST are complex and scanning on high-field MRI is required to be able to detect the small difference between the signal of bulk water protons and GAG bound protons.^20^

Collagen network integrity is measured by T2 mapping. Disruption of the collagen structure increases the mobility of protons and therefore produces higher T2 relaxation times. Furthermore, the well-organized structure of collagen matrix in healthy cartilage gives rise to a zonal difference in T2 relaxation times between the deep layer and the superficial layer which is absent in degenerated cartilage.^21^

The first aim of this study was to evaluate the morphological and biochemical quality of cartilage transplants and the status of the articular cartilage of patients 25 years after PT and ACT as measured by ultra-high-field 7T MRI and to present these findings next to clinical outcome. The second aim was to assess intralesional osteophyte formation of the transplants and evaluate its effect on the quality of opposing tibial cartilage, as measured by 7T MRI.

## Materials and Methods

### Patient Population

Perichondrium transplantation patients and ACT patients who underwent surgery between 1986 and 1996 were included from 2 different databases. The PT database consisted of 88 Dutch patients and the ACT database consisted of 400 Swedish patients. To optimize the comparison of the cartilage tissue, only patients with a repaired cartilage defect on the femoral condyle were included. Furthermore, for Dutch PT patients specifically: they needed to be willing to visit the outpatient clinic and undergo a 7T MRI scan in Maastricht; for Swedish ACT patients specifically: they needed to be willing to travel to the Netherlands and undergo a 7T MRI scan in Maastricht. All participants had to approve that coincidental findings would be reported to their general practitioner and approve storing and use of their data for research purposes. Exclusion criteria were knee arthroplasty in the area of the transplant (i.e., total-, hemi-knee arthroplasty); major surgery of transplant in the knee (e.g., patellectomy and microfracture); severe OA (e.g., grade-4 Kellgren and Lawrence classification); contra-indications for 7T MRI scanning. The in- and exclusion criteria, combined with our very long-term follow-up in which a considerable number of patients developed severe OA caused eligibility for only 12 patients to be enrolled in our study.

Perichondrium transplantation patients were notified of plans to perform 7T MRI scanning of the transplants for research purposes at the time of participation in the long-term follow-up study of PT.^8^ An information letter to explain the study was sent to eligible patients. A week thereafter, the patients were contacted by phone by the research physician (M.J.) to answer questions if any and to ask whether they were willing to participate in the study. Eligible ACT patients were contacted by phone by their surgeon (L.P.) to explain the study and to ask whether they were willing to participate.

This study was performed in accordance with the Helsinki Declaration of 1975, as revised in 2013, and the protocol was accepted by the medical ethical committee of the Maastricht University Medical Center (NL48277.068.14/METC 142039) in which patients gave their written informed consent. Participants from Sweden signed a certified, translated version of the written informed consent, translated by Metamorfose Vertalingen, Utrecht, the Netherlands.

### Surgical Procedures

A comprehensive description of the surgical procedures has been reported before by Homminga *et al.*, Bouwmeester *et al.* for PT, and by Peterson *et al.* for ACT.^5,7,9,10,22^ In short, PT is a one-stage procedure. A piece of perichondrium was dissected from the cartilaginous part of one of the lower ribs and removed together with its cambium layer. The graft was cut to match the size of the defect. Subsequently, the perichondral graft was placed in the defect with the chondral side facing up and attached with fibrin glue.^5,10^

Autologous chondrocyte transplantation includes 2 surgical procedures. During the first surgical procedure, cartilage tissue was harvested from a healthy, nonweight-bearing part of the cartilage. From this tissue, chondrocytes were retrieved and cultured in a laboratory for several weeks. During the second surgical procedure, the chondrocytes from the cell culture were injected into the defect under a periosteal flap.^9^

### Clinical Questionnaires/Patient-Reported Outcome Measures

Patients were asked to complete 3 clinical questionnaires at the time of MRI acquisition: the International Knee Documentation Committee (IKDC),^23^ the Knee injury and Osteoarthritis Outcome Score (KOOS)^24^ (Validated Swedish version),^25^ and the Visual Analogue Scale (VAS) for knee pain.

### MRI Acquisition

Morphological and biochemical MRI measurements were performed on a 7T MR whole body system (Magnetom, Siemens Healthcare, Erlangen, Germany) using a 28-channel proton knee coil (QED, Electrodynamics LLC, Cleveland, OH). Before acquiring MRI data, the homogeneity of the main magnetic field (B_0_) was optimized by a B_0_ shim. The radiofrequency pulse (B_1_) was optimized by acquiring a B_1_ map. To avoid motion artifacts, the leg was stabilized using a vacuum cushion underneath the lower leg.

The morphological protocol included a 3-dimensional (3D) T2 dual-echo steady-state (DESS) sequence. The T2 DESS sequence was obtained for the complete knee in sagittal plane. Furthermore, a 2-dimensional (2D) sagittal proton-density (PD) weighted fast spin-echo (FSE) sequence with fat suppression (fatsat) was obtained. The biochemical protocol included T2 mapping and gagCEST sequences.

The T2 relaxation times were obtained from T2 maps that were reconstructed using a multi-echo, spin-echo technique, using a custom written Matlab script.^26^ The T2 mapping protocol was obtained in sagittal direction. Due to SAR restrictions, only the femoral condyle containing the cartilage repair tissue region was acquired. The obtained T2 relaxation times are a measure for collagen integrity: the higher the T2 relaxation time, the lower the integrity of the collagen network.^21^

For gagCEST imaging, a 3D radiofrequency (RF) spoiled gradient echo (GRE) sequence including 19 saturation RF pulses was acquired. One additional measurement without the presaturation pulses was acquired. Residual transversal magnetization signal was spoiled by gradient spoiling. The applied B_1_ amplitude of the saturation pulses was set to a minimum of 0.8 µT and adapted for each individual to the maximum value possible in relation to SAR, to achieve optimal saturation. The separate saturation measurements were postprocessed into colored GAG maps using a custom made Matlab script which determined the magnetization transfer ratio asymmetry (MTRasym) in the calculated *z*-spectra.^20^ The MTRasym value is a measure for GAG content: the higher the MTRasym value, the higher the GAG content.^19^ Imaging parameters for the morphological and biochemical sequences are presented in **Table 1**.

**Table 1.** Imaging Parameters for Morphological Sequences T2 DESS and PD Fatsat FSE and for Biochemical Sequences T2 mapping and gagCEST.

	T2 DESS	PD Fatsat FSE	T2mapping	gagCEST
Repetition time (ms)	8.90	7,440	2,200	6.90
Echo time (ms)	2.63	36	13.8, 27.6, 41.4, 55.2, 69.0, 82.8	2.84
Flip angle (°)	18	180	180	9
Field of view (mm^2^)	160 × 160	160 × 160	136 × 160	157 × 180
Matrix size	320 × 320	864 × 864	320 × 272	192 × 168
Voxel size (mm^3^)	0.5 × 0.5 × 0.5	0.4 × 0.4 × 2.5	0.5 × 0.5 × 3.0	0.9 × 0.9 × 2.2
Acceleration factor (GRAPPA)	3	3	2	2
Acquisition time (min)	05:00	08:42	10:57	20:04

DESS = dual echo steady state; PD = proton-density; FSE = fast spin-echo; gagCEST = glycosaminoglycan Chemical Exchange Saturation Transfer.

### MRI Analysis

The morphological MR data sets were transferred to a freeware JiveX imaging viewer (VISUS Technology Transfer GmbH, Bochum, Germany). The Magnetic Resonance Observation of Cartilage Repair Tissue (MOCART)^27^ was used to assess the cartilage transplant tissue and was scored together with the cartilage quality in the rest of the joint by the senior author (S.T.; radiologist with over 25 years of experience in musculoskeletal imaging), in consensus with a resident orthopedic surgeon (M.J.). In case of any uncertainties, an experienced orthopedic surgeon with over 10 years of experience in cartilage repair of the knee (P.E.) was consulted.

**Figure 1.**
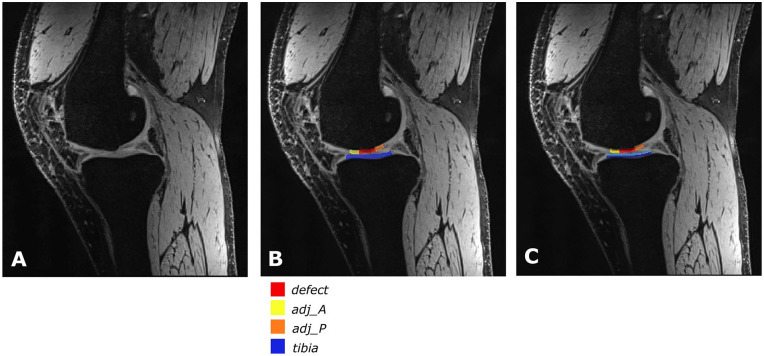
Example of ROIs in a slice with defect: (**A**) original image, T2 DESS, (**B**) image with ROIs to obtain MTRasym and global T2 relaxation times: defect ROI in red (defect); anterior adjacent ROI in yellow (adj_A); posterior adjacent ROI in orange (adj_P); tibia ROI in blue (tibia), and (**C**) image with ROIs divided in a deep zone (dark colors) and a superficial zone (light colors) to obtain zonal T2 relaxation times. ROI = region of interest; DESS = dual echo steady state; MTRasym = magnetization transfer ratio asymmetry.

**Figure 2.**
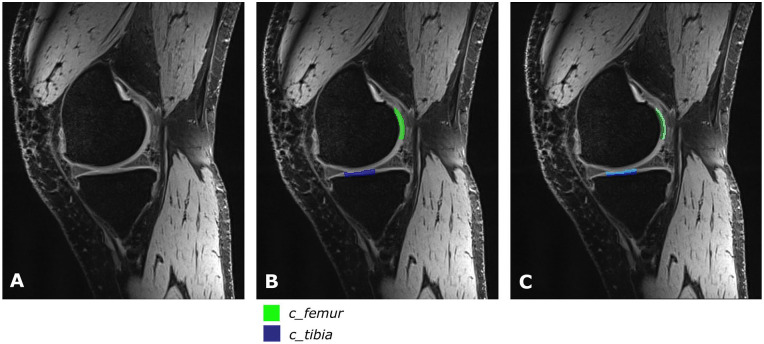
Example of ROIs in a control slice: (**A**) original T2 DESS image and (**B**) image with control ROIs to obtain MTRasym and global T2 relaxation times. Control region in the femur is presented in green (c_femur), control region for the tibia from meniscus to meniscus is presented in blue (c_tibia). (**C**) Image with control ROIs divided in a deep zone (dark colors) and a superficial zone (light colors) to obtain zonal T2 relaxation times. ROI = region of interest; DESS = dual echo steady state; MTRasym = magnetization transfer ratio asymmetry.

The morphological MR data sets as well as the postprocessed biochemical T2 maps and GAG maps were transferred to OsiriX imaging software (v.9.0.2, Pixmeo, Switzerland) and analyzed based on a region of interest (ROI) approach. The resolution of the biochemical T2 maps and GAG maps were adapted to the resolution of the morphological DESS images by linear interpolation. As the images were acquired in the same plane, only translation according to their DICOM tags was necessary to register the images. No motion correction was applied; however, the overlay was manually checked by comparing anatomical landmarks in both sequences and adjusted when deemed necessary. Regions of interest were manually drawn in the DESS morphological image of each patient by 2 independent readers (M.J. and M.P.), the ROIs were finalized after consensus. The inclusion of cartilage pixels only was ensured; no bone pixels or joint fluid pixels were included in the ROIs. Per patient, regions were selected in a slice showing the defect clearly (**Figure 1**) and regions were selected in a control slice (**Figure 2**). Six ROIs were drawn per patient; a defect ROI (referred to as *defect*) with anterior and posterior adjacent ROIs in the femur (referred to as *adj_A* and *adj_P*, respectively); an ROI in the tibia cartilage opposite to the defect between the area covered by the meniscus (referred to as *tibia*); a control ROI in the posterior part of the femur (referred to as *c_femur*); and a control tibia ROI (referred to as *c_tibia*). The ROIs were subsequently transferred to the coregistered GAG maps and T2 maps. In case of the GAG maps, the mean MTRasym value within each ROI was extracted (see **Figs. 1B** and **2B**) as a measure of GAG content. In case of the T2 maps, the mean T2 relaxation time within each ROI as a whole (global T2 relaxation time) as well as within the deep zone and the superficial zone specifically (deep zone T2 relaxation time and superficial zone T2 relaxation time, respectively) were extracted (see **Figs. 1C** and **2C**), as a measure for the collagen integrity.

### Calcification Thickness

**Figure 3.**
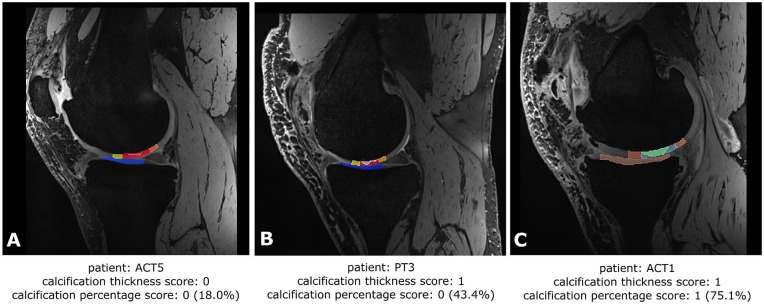
Examples of the calcification scoring with the calcification presented in white: (**A**) a calcification covering 18% of the defect with a substantial layer of cartilage between the calcification and the opposing tibial cartilage, (**B**) a calcification covering less than half of the defect with contact of the calcification with the opposing tibial cartilage, and (**C**) a calcification covering more than half of the defect with no layer of cartilage between the calcification and the opposing tibial cartilage. ACT = autologous chondrocyte transplantation; PT = perichondrium transplantation.

Calcification was scored in the T2 DESS morphological image of each patient by 2 independent readers (M.J. and M.P.), the ROIs were finalized after consensus. The used technique is an adaptation from the technique used by Demange and colleagues.^13^ An ROI was drawn that included the calcified area of the graft. The *percentage of calcification* was calculated by dividing the number of calcified pixels within the graft by the number of pixels in the total graft. Subsequently, patients with a calcification percentage of less than 50% were given calcification percentage score 0, patients with a calcification percentage of more than 50% were given calcification percentage score 1. Furthermore, the *thickness of the calcification* was scored. Patients with a calcification penetrating the surface of the cartilage layer, thus with the calcification being in direct contact with the opposing tibial cartilage, were given a calcification thickness score of 1. Patients with a calcification that was still covered by a layer of cartilage (regardless of the thickness of that layer of cartilage) preventing direct contact between the calcification and the opposing tibial cartilage were given a calcification thickness score of 0. Examples of the calcification scores are provided in **Figure 3**. Subsequently, the influence of the calcification of the cartilage grafts was compared to the quality of the opposing cartilage tissue.

### Statistical Analysis

Statistical analysis was performed using IBM SPSS statistics, version 25 (IBM, Armonk, New York). Normality was tested by a Shapiro-Wilk test. Differences between PT and ACT patients were assessed by an independent *t*-test in case of normality and a Mann-Whitney *U*-test otherwise. Differences between regions were evaluated by a paired samples *t*-test in case of normality and a Wilcoxon Signed-Rank test otherwise. Differences were considered statistically significant when the *P* value was below .05.

## Results

### Description of Patient Population

Seven PT patients and 5 ACT patients were willing to be included in the study. Baseline demographics are provided in **Table 2**. Time between surgery and MRI follow-up was similar for the PT patients and the ACT patients, on average 28.1 years for PT and 24.0 years for ACT (*P* value .213). Defect size was larger in the ACT patients compared to the PT patients (3.9 cm^2^ and 2.1 cm^2^, respectively, *P* value .048). No adverse events or serious adverse events occurred during this study.

**Table 2.** Patient Characteristics for the Dutch PT Patients (PT1-PT7) and the Five Swedish ACT Patients (ACT1-ACT5) Including Mean Values, Standard Deviation (SD) and *P* Values for the Numeric Characteristics.

	Sex	Age at Surgery (years)	BMI (kg/m^2^)	Knee	Location Defect	Defect Size (cm^2^)	Follow-Up Duration (years)
PT1	Male	36	27.5	Right	MFC	2.3	24
PT2	Female	22	23.8	Right	MFC	0.5	25
PT3	Male	45	29.4	Right	MFC	0.8	30
PT4	Male	35	26.3	Left	MFC	2.3	31
PT5	Female	17	23.0	Left	MFC	3.0	29
PT6	Male	23	22.8	Right	MFC	3.0	29
PT7	Male	27	29.1	Left	MFC	3.1	29
**Mean**	**-**	**29.3**	**26.0**		**-**	**2.1**	**28.1**
**SD**	**-**	**9.8**	**2.8**		**-**	**1.1**	**2.6**
ACT1	Male	24	32.1	Left	MFC	2.0	30
ACT2	Male	27	24.3	Right	LFC	3.0	30
ACT3	Male	32	29.0	Right	MFC	5.2	24
ACT4	Male	28	27.5	Right	MFC	3.3	11
ACT5	Male	27	27.5	Right	MFC	6.0	25
**Mean**	**-**	**27.6**	**28.0**	**-**	**-**	**3.9**	**24.0**
**SD**	**-**	**2.9**	**2.8**	**-**	**-**	**1.6**	**7.8**
***P* value**		**.719**	**.235**			**.048**	**.213**

PT = perichondrium transplantation; ACT = autologous chondrocyte transplantation; kg/m^2^ = kilograms per square meter; cm^2^ = square centimeter; SD = standard deviation; MFC = medial femoral condyle; LFC = lateral femoral condyle.

### Clinical Outcome at Time of MRI

The IKDC, KOOS, and VAS questionnaire scores of each individual patient at the time of MRI acquisition are presented in **Table 3**. No statistically significant difference was found between the questionnaire scores of the PT patients and the ACT patients.

**Table 3.** Individual Scoring Parameters Including the IKDC, KOOS and VAS Questionnaire Scores Were Used to Assess Clinical Outcome.

Patient Number	IKDC	KOOS					VAS	MOCART Score	Cartilage Quality in the Rest of the Joint
		Pain	Other Symptoms	Function in Daily Living	Function in Sport and Recreation	Knee-Related Quality of Life			
PT1	86.2	94.4	75.0	100.0	100.0	100.0	5	65	Moderate degeneration
PT2	60.9	84.4	92.9	95.6	75.0	68.8	20	85	Early degeneration
PT3	26.4	16.7	50.0	16.2	0.0	0.0	85	75	Early-moderate degeneration
PT4	88.5	100.0	100.0	100.0	100.0	100.0	5	80	Early degeneration
PT5	73.6	100.0	96.4	100.0	90.0	83.3	0	65	Early degeneration
PT6	75.9	100.0	82.1	100.0	80.0	75.0	0	85	Early-moderate degeneration
PT7	34.5	25.0	28.6	22.1	0.0	12.5	80	60	Early-severe degeneration
**Mean**	**63.7**	**74.4**	**75.0**	**76.3**	**63.6**	**63.8**	**27.9**	**73.6**	**N.A.**
**SD**	**24.6**	**37.1**	**26.6**	**39.1**	**44.4**	**40.5**	**38.0**	**10.3**	**N.A.**
ACT1	79.3	88.9	53.6	97.1	80.0	56.3	10	80	Early-severe degeneration
ACT2	43.7	77.8	39.3	70.6	35.0	50.0	30	55	Early-severe degeneration
ACT3	74.7	75.0	60.7	92.6	35.0	87.5	0	80	Early-moderate degeneration
ACT4	72.4	91.7	78.6	83.8	45.0	62.5	20	65	Early degeneration
ACT5	80.5	97.2	89.3	100.0	90.0	93.8	30	75	Early-severe degeneration
**Mean**	**70.1**	**86.1**	**64.1**	**88.8**	**57.0**	**70.0**	**18.0**	**71.0**	**N.A.**
**SD**	**15.1**	**9.4**	**19.9**	**11.9**	**26.1**	**19.5**	**13.8**	**10.8**	**N.A.**
***P* value**	**.867**	**.639**	**.432**	**.639**	**.639**	**.876**	**.755**	**.639**	**N.A.**

The MRI-based MOCART score was used to assess the cartilage transplant quality and was scored together with the cartilage quality in the rest of the joint by the senior author (S.T.). Overall cartilage quality was divided in the categories: no degeneration, early degeneration, moderate degeneration and severe degeneration. Mean values with standard deviation (SD) for the PT patients and the ACT patients were included.

IKDC = International Knee Documentation Committee; KOOS = Knee injury and Osteoarthritis Outcome Score; VAS = Visual Analogue Scale; MOCART = Magnetic Resonance Observation of Cartilage Repair Tissue; PT = perichondrium transplantation; N.A. = Not applicable; SD = standard deviation; ACT = autologous chondrocyte transplantation.

### Morphological Assessment and MOCART Score

The morphological MR images of the 7 PT patients and the 5 ACT patients were available for assessment of the transplant by means of the MOCART score. The outcome of the 10 MOCART criteria per patient are presented in the **Supplemental Table 1**. The overall MOCART score and the cartilage quality in the rest of the joint are presented in **Table 3** for each individual patient. The cartilage quality in the rest of the joint was varying from no degeneration to severe degeneration. The overall MOCART score was similar for the PT patients and the ACT patients (mean score 73.6 and 71.0 respectively, *P* value = .639).

Evaluation of the graft alone showed similar intralesional osteophyte formation in the perichondrium transplants compared to the autologous chondrocyte transplants (**Table 4**). In 5 of the 12 patients, the grafts were calcified more than 50%, and also in 5 of the 12 patients the calcification penetrated the surface of the graft.

**Table 4.** Calcification Scores.

Patient	Calcification Percentage		Calcification Thickness Score
		Score	
PT1	25.9%	0	0
PT2	47.9%	0	0
PT3	43.4%	0	1
PT4	47.0%	0	0
PT5	57.4%	1	1
PT6	73.6%	1	1
PT7	57.4%	1	0
ACT1	75.1%	1	1
ACT2	31.1%	0	0
ACT3	92.4%	1	1
ACT4	22.0%	0	0
ACT5	18.0%	0	0

PT = perichondrium transplantation; ACT = autologous chondrocyte transplantation.

### Biochemical Assessment

**Figure 4.**
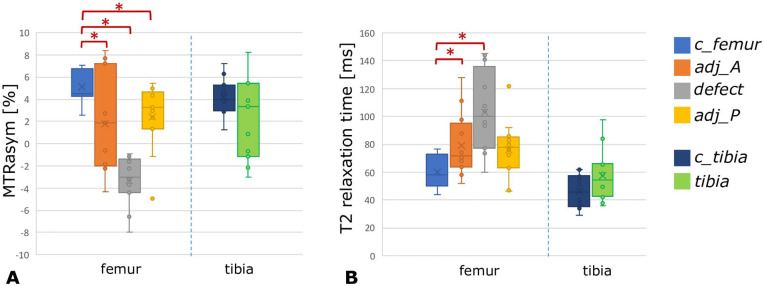
MTRasym values (**A**) and global T2 relaxation times (**B**) for the 6 different ROIs. The regions in the femur are displayed on the left side of the dotted line, and regions in the tibia are presented on the right side of the dotted line. Red asterisks represent statistically significant differences between regions. MTRasym = magnetization transfer ratio asymmetry.

**Figure 4** shows an overview of the biochemical values of cartilage in the specified ROIs. The biochemical values for each of the 6 regions are presented next to the overall MOCART score per patient in **Supplemental Table 2**. Paired samples *t*-test showed that GAG content in the defect region as well as in the adjacent regions was significantly lower than the GAG content in the control ROI. The MTRasym value in the tibia cartilage opposing the defect was similar to the MTRasym value of control tibia cartilage, suggesting similar GAG content in both regions. Paired samples *t*-test showed significantly higher global T2 relaxation times for the defect region and for anterior adjacent region, compared to the femur control region. The global T2 relaxation times in the tibia region opposing the defect were similar to global T2 relaxation times in the control tibia cartilage, suggesting similar collagen integrity for both regions.

**Figure 5.**
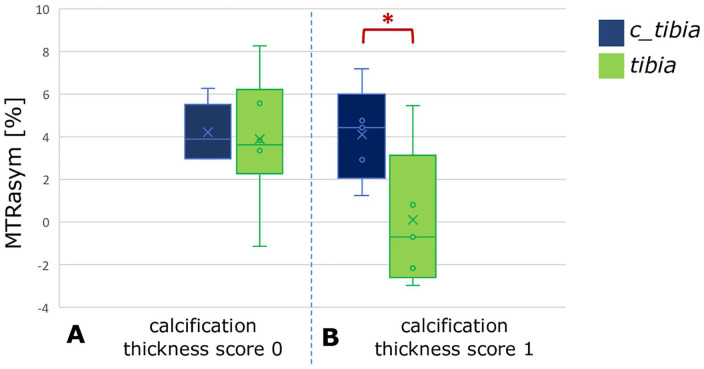
MTRasym values for the tibial cartilage opposing the defect (tibia) compared to control tibial cartilage (c_tibia) for patients with calcification thickness score 0 (**A**) and for calcification thickness score 1 (**B**). The red asterisk represents a statistically significant difference between regions. MTRasym = magnetization transfer ratio asymmetry.

**Figure 6.**
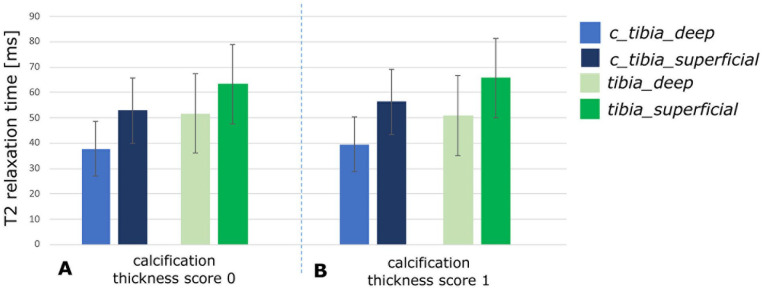
T2 relaxation times for the tibial cartilage opposing the defect (tibia) compared to control tibial cartilage (c_tibia) for the calcification thickness score 0 (**A**) and for the calcification thickness score 1 (**B**) in the deep zone and in the superficial zone of the cartilage.

**Table 4** presents the calcification scores for the included patients. The influence of calcification thickness of the transplant on the opposing cartilage is presented in **Figure 5** for the gagCEST sequence and in **Figure 6** for T2 mapping. Statistical analysis showed that the tibial cartilage opposing the defect in patients with a calcification that is in contact with the opposing cartilage (calcification thickness score of 1) has significantly lower GAG content (MTRasym value) compared to control tibial cartilage, while the collagen integrity (global T2 relaxation times) was similar for tibial cartilage opposing the defect and control tibial cartilage. **Figure 6** shows that the zonal variation between the deep layer and the superficial layer of the tibial cartilage opposing the defect is similar to that of the control tibia cartilage. In other words, the collagen integrity of the opposing cartilage was not affected by the calcification thickness of the transplant.

**Figure 7.**
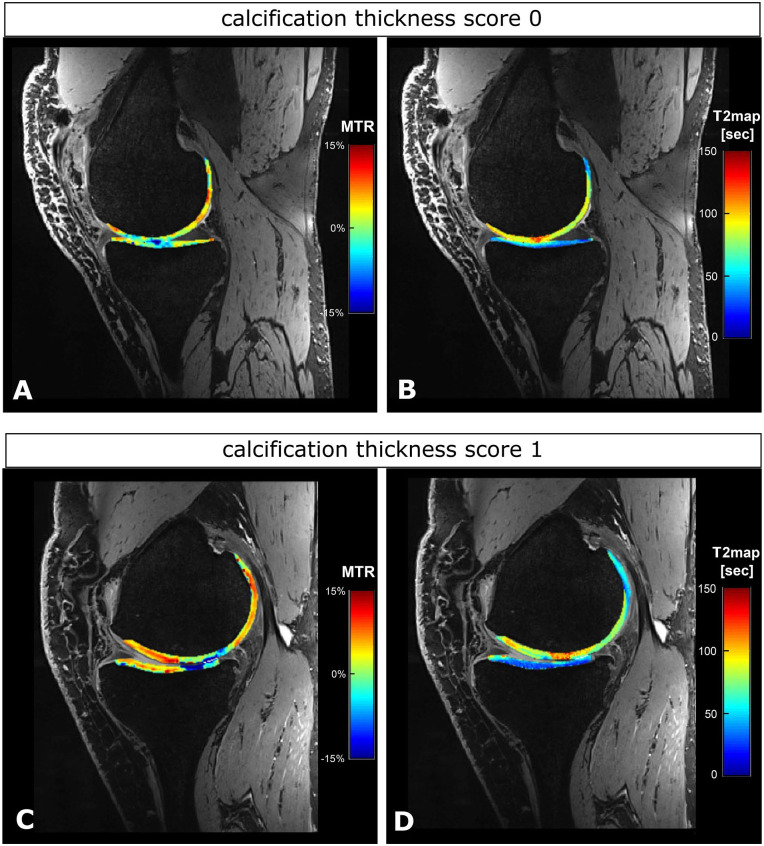
Example of a patient with calcification thickness score 0 (**A** and **B**) and a patient with calcification thickness score 1 (**C** and **D**). DESS images are presented with an overlay of MTRasym values and T2 relaxation times. DESS = dual-echo steady state; MTRasym = magnetization transfer ratio asymmetry.

**Figure 7** illustrates the findings of **Figures 4** to **6** in the form of MTR asymmetry and T2 relaxation time overlays for a patient with calcification thickness score of 0 and for a patient with calcification thickness score 1. For both patients, the transplant area shows low MTRasym values and high T2 relaxation times compared to control regions, indicating a lower GAG content and a disturbed collagen network. For the patient with cartilage thickness score 0 (**Fig. 7A** and **B**), the opposing cartilage is of relatively good quality. The patient with a calcification thickness score of 1 (**Fig. 7C** and **D**) showed lower GAG content in the opposing cartilage while the collagen integrity does not seem to be influenced.

## Discussion

In this study, 12 patients were evaluated about 25 years after cartilage repair surgery of the knee by means of clinical questionnaires and 7T MRI. The cartilage tissue in general, the cartilage repair tissue and the opposing tibial cartilage were assessed both morphologically and biochemically by 7T MRI. The quality of the cartilage tissue throughout the joint was variable. For each of the included cartilage repair patient, the cartilage repair tissue was of poor quality (low GAG content [low MTRasym values] and low collagen integrity (high T2 relaxation times)), regardless of the performed procedure. In line with previous research, we found a high incidence of intralesional osteophytes. The thickness of the calcification in these intralesional osteophytes can influence the opposing tibial cartilage. It was shown that when the intralesional osteophyte penetrates the surface of the graft, the opposing tibial cartilage was biochemically damaged. The damage was more pronounced in the GAG content reflected by the MTRasym values and less in the collagen integrity represented by the intact zonal variation in T2 relaxation times, suggesting that tibial cartilage opposing osteophytes that penetrate the surface showed signs of early OA. A difference in percentage of calcification of the grafts caused no statistically significant difference of opposing cartilage tissue quality. Calcified tissue has an increased stiffness compared to cartilage, which causes higher contact stresses and increased friction.^28^ This increased stiffness and friction of a calcification that penetrates the surface of a graft is expected to exert a larger mechanical strain on the opposing tibial cartilage compared to an intact surface and thereby causing its deterioration over time. The 10- to 20-year clinical outcome of cartilage repair surgery has been documented previously by multiple authors, for example by Minas and co-workers with satisfactory results.^29-31^ However, to our knowledge, there are no studies that describe the biochemical status of the articular cartilage of patients after a follow-up of a mean of 25 years as described in this paper. Evaluation of articular cartilage by 7T MRI provides the opportunity for its biochemical assessment and a high spatial resolution for detailed morphological assessment. So far, the evaluation of the GAG content in repair tissue, an important marker for the biomechanical properties was restricted to dGEMRIC at lower field MR,^32^ which however requires a double dose of intravenous administration of Gadolinium-based contrast agents, which considering the ongoing discussions of Gadolinium depositions in the brain are problematic. In addition, the standard ionic contrast agent so far used for dGEMRIC, Magnevist, was removed from the European market by the European Medicine Ageny due to the Gadolinium depositions in the human body seen with linear Gadolinium-based contrast agents.^33^ High spatial resolution can be achieved using new 3T MRI techniques and T2 mapping is available at 3T MRI as well as on 7T MRI, but gagCEST is limited to use at ultra-high-field such as 7T MRI.^20^ Using gagCEST, the GAG content can be quantified using a regular proton coil (no sodium coil needed) and without the use of a contrast agent (as is the case for dGEMRIC). On the downside, gagCEST is limited to high-field MRI such as 7T MRI, because the spectral resolution on 7T is by a factor of 2 higher compared to 3T, which is needed to separate the small frequency shift between protons bound to GAG and protons in the water pool.^20^ Therefore, gagCEST scanning is only feasible at 7T MRI and provides essential biochemical information not available in studies performed with lower field MRI (1.5T or 3.0T).

The occurrence of intralesional osteophytes after cartilage repair surgery has been described before, the incidence of osteophytes rises when the subchondral bone is involved in either the defect or the surgery.^13,14,34^ Intralesional osteophytes occur more often after ACT procedures with previous marrow stimulation and in periosteal-covered defects compared to collagen membrane-covered defects,^13^ but it is still unclear whether the osteophytes result from a thickening of the subchondral bone or from the progenitor cells in the cambium layer of the periosteal tissue.^35^ Kreuz *et al.* propose an impaired clinical outcome after microfracture caused by a thinner layer of cartilage overlying damaged subchondral bone and subsequent increased shear stresses.^34^ However, calcification of the repair tissue was not a part of the MRI scoring systems at the time of publication, nor was calcification described separately in their paper.^34^ In addition, Pestka *et al.* describe an increased failure rate after previous marrow stimulation but did not directly correlate this to increased intralesional osteophytes.^36^

In a review focusing on the subchondral bone in osteochondral repair, Orth *et al.* elucidate on the lack of detailed visualization of subchondral bone architecture of repaired cartilage due to technical and ethical limitations. Although there is no absolute lack of studies which assess the repaired cartilage morphologically (often by MRI), a detailed biochemical assessment of repair tissue and evaluation of intralesional osteophytes is less common.^37^ Recently, the MOCART score has been updated to provide a more detailed assessment of morphologic characteristics of the repaired cartilage resulting in the MOCART 2.0 score;^38^ however, at this moment, it has only been applied in 3 clinical cartilage repair studies.^39-41^ Only Sessa *et al.* found a correlation of the MOCART 2.0 score with clinical outcome parameters. However, group sizes in these studies were relatively small and therefore might lack the statistical power to detect correlations.^39-41^ Even though our study also assessed a relatively small group of patients, we did find a correlation of surface penetrating intralesional osteophytes which led to opposing cartilage damage. Based on our current data, we are not able to demonstrate an impaired subjective or clinical outcome caused by intralesional osteophyte formation after cartilage repair surgery.

An important limitation of this study was the small sample size. The small numbers of patients for the 2 surgical procedures did not allow for a long-term comparison between the procedures. The heterogeneity among the included patients was another limitation. Some patients underwent reconstruction of their anterior cruciate ligament in combination with cartilage repair of their defect. Furthermore, it was difficult to select control ROIs in the knees of the patients given that 25 years after surgery, the quality of the knee cartilage was in general relatively low. In addition, 7T MRI has only been obtained at a long-term follow-up. To demonstrate the value of clinical evaluation of articular cartilage repair surgery by 7T MRI, larger group sizes and monitoring over several timepoints should be included in future work. It is important to note that ACT has been modified since 1996 to stop the intralesional osteophyte formation by careful removal of the calcified layer down to the subchondral bone plate, release the tourniquet to detect and stop any bleeding by fibrin glue. Furthermore, the periosteal flap has been replaced by synthetical resorbable membranes.

To conclude, PT and ACT patients have a high incidence of intralesional osteophyte formation 25 years after surgery. The resulting biochemical damage to the opposing tibial cartilage might be dependent on osteophyte morphology.

## Supplemental Material

sj-docx-1-car-10.1177_19476035211060506 – Supplemental material for 7-Tesla MRI Evaluation of the Knee, 25 Years after Cartilage Repair Surgery: The Influence of Intralesional Osteophytes on Biochemical Quality of Cartilage Click here for additional data file. Supplemental material, sj-docx-1-car-10.1177_19476035211060506 for 7-Tesla MRI Evaluation of the Knee, 25 Years after Cartilage Repair Surgery: The Influence of Intralesional Osteophytes on Biochemical Quality of Cartilage by M.P.F. Janssen, M.J.M. Peters, E.G.M. Steijvers-Peeters, P. Szomolanyi, E.M.C. Jutten, L.W. van Rhijn, L. Peterson, A. Lindahl, S. Trattnig and P.J. Emans in CARTILAGE

sj-docx-2-car-10.1177_19476035211060506 – Supplemental material for 7-Tesla MRI Evaluation of the Knee, 25 Years after Cartilage Repair Surgery: The Influence of Intralesional Osteophytes on Biochemical Quality of Cartilage Click here for additional data file. Supplemental material, sj-docx-2-car-10.1177_19476035211060506 for 7-Tesla MRI Evaluation of the Knee, 25 Years after Cartilage Repair Surgery: The Influence of Intralesional Osteophytes on Biochemical Quality of Cartilage by M.P.F. Janssen, M.J.M. Peters, E.G.M. Steijvers-Peeters, P. Szomolanyi, E.M.C. Jutten, L.W. van Rhijn, L. Peterson, A. Lindahl, S. Trattnig and P.J. Emans in CARTILAGE
