# Supplementary material for: 7-Tesla MRI Evaluation of the Knee, 25 Years after Cartilage Repair Surgery: The Influence of Intralesional Osteophytes on Biochemical Quality of Cartilage
Source: Cartilage. 2021 Nov 26;13(1 Suppl):767S–779S. doi: 10.1177/19476035211060506 (PMC8808805; doi:10.1177/19476035211060506)
Supplement: sj-docx-1-car-10.1177_19476035211060506 – Supplemental material for 7-Tesla MRI Evaluation of the Knee, 25 Years after Cartilage Repair Surgery: The Influence of Intralesional Osteophytes on Biochemical Quality of Cartilage [file sj-docx-1-car-10.1177_19476035211060506.docx]

**Supplemental Table 1.** Patient individual scores of MOCART variable.

| Patient number | Degree of repair and filling of the defect | Integration to border zone | Surface of the repair tissue | Structure of the repair tissue | Signal intensity of the repair tissue | | Subchondral lamina | Subchondral bone | Adhesions | Effusion | MOCART score |
| --- | --- | --- | --- | --- | --- | --- | --- | --- | --- | --- | --- |
|  |  |  |  |  | 2D PD fatsat FSE | 3D  T2 DESS |  |  |  |  |  |
|  | | | | | | | | | | | |
| PT1 | 15 | 15 | 10 | 0 | 5 | 5 | 5 | 0 | 5 | 5 | 65 |
| PT2 | 15 | 15 | 10 | 0 | 15 | 15 | 5 | 0 | 5 | 5 | 85 |
| PT3 | 20 | 15 | 10 | 0 | 15 | 5 | 5 | 0 | 5 | 0 | 75 |
| PT4 | 20 | 10 | 5 | 0 | 15 | 15 | 5 | 0 | 5 | 5 | 80 |
| PT5 | 20 | 10 | 5 | 0 | 5 | 15 | 5 | 0 | 5 | 0 | 65 |
| PT6 | 15 | 15 | 10 | 0 | 15 | 15 | 5 | 0 | 5 | 5 | 85 |
| PT7 | 20 | 15 | 5 | 0 | 5 | 5 | 5 | 0 | 5 | 0 | 60 |
| **mean** | **17.9** | **13.6** | **7.9** | **0** | **10.7** | **10.7** | **5.0** | **0.0** | **2.0** | **2.9** | **73.6** |
| **SD** | **2.7** | **2.4** | **2.7** | **0** | **5.3** | **5.3** | **0.0** | **0.0** | **0.0** | **2.7** | **10.3** |
|  | | | | | | | | | | | |
| ACT1 | 20 | 10 | 10 | 0 | 15 | 15 | 5 | 0 | 5 | 0 | 80 |
| ACT2 | 10 | 10 | 10 | 0 | 15 | N.A. | 5 | 0 | 5 | 0 | 55 |
| ACT3 | 10 | 15 | 10 | 5 | 15 | 15 | 5 | 0 | 5 | 0 | 80 |
| ACT4 | 10 | 15 | 5 | 0 | 15 | 5 | 5 | 0 | 5 | 5 | 65 |
| ACT5 | 10 | 15 | 10 | 0 | 15 | 15 | 5 | 0 | 5 | 0 | 75 |
| **mean** | **12.0** | **13.0** | **9.0** | **1.0** | **15.0** | **12.5** | **5.0** | **0.0** | **5.0** | **1.0** | **71.0** |
| **SD** | **4.5** | **2.7** | **2.2** | **2.2** | **0** | **5.0** | **0.0** | **0.0** | **0.0** | **2.2** | **10.8** |
|  |  |  |  |  |  |  |  |  |  |  |  |
| **p-value** | **0.048** | **0.755** | **0.530** | **0.639** | **0.268** | **0.648** | **1.000** | **1.000** | **1.000** | **0.343** | **0.639** |
