# Supplementary material for: 7-Tesla MRI Evaluation of the Knee, 25 Years after Cartilage Repair Surgery: The Influence of Intralesional Osteophytes on Biochemical Quality of Cartilage
Source: Cartilage. 2021 Nov 26;13(1 Suppl):767S–779S. doi: 10.1177/19476035211060506 (PMC8808805; doi:10.1177/19476035211060506)
Supplement: sj-docx-2-car-10.1177_19476035211060506 – Supplemental material for 7-Tesla MRI Evaluation of the Knee, 25 Years after Cartilage Repair Surgery: The Influence of Intralesional Osteophytes on Biochemical Quality of Cartilage [file sj-docx-2-car-10.1177_19476035211060506.docx]

**Supplemental Table 2.** For each patient the overall MOCART score is presented next to the biochemical MRI measures for collagen integrity (T2 relaxation time) and GAG content (MTR asymmetry) for the four regions of interest in the defect slice (adjA= adjacent region anterior to the defect; defect = defect region; adjP = adjacent region posterior to the defect; tibiamen = region in the tibia cartilage opposing the defect) and for the two regions of interest in the control slice (femur = control region in the femur cartilage; tibiamen = control region in the tibia cartilage opposing the defect).

|  |  | MOCART | T2 relaxation time [ms] | | | | | | MTR asymmetry [%] | | | | | |
| --- | --- | --- | --- | --- | --- | --- | --- | --- | --- | --- | --- | --- | --- | --- |
|  |  |  | defect slice | | | | control slice | | defect slice | | | | control slice | |
|  |  |  | adjA | defect | adjP | tibiamen | femur | tibiamen | adjA | defect | adjP | tibiamen | femur | tibiamen |
|  |  |  |  |  |  |  |  |  |  |  |  |  |  |  |
| NL1 | PT1 | 65 | 57,78 | 59,67 | 72,31 | 37,83 | 49,76 | 57,66 | 6,31 | -2,54 | 4,36 | 1,83 | 4,71 | 5,29 |
| NL2 | PT2 | 85 | 111,46 | 144,19 | 121,77 | 84,19 | 43,89 | 40,51 | -2,25 | -0,95 | 5,40 | 3,86 | 4,26 | 3,80 |
| NL3 | PT3 | 75 | 87,49 | 103,81 | 85,88 | 49,88 | 50,87 | 61,76 | -0,64 | -3,27 | 1,35 | -2,16 | 2,60 | 1,22 |
| NL4 | PT4 | 80 | 74,38 | 107,30 | 63,84 | 49,31 | 74,35 | 56,76 | -4,29 | -3,71 | 3,56 | 8,26 | 5,10 | 6,27 |
| NL5 | PT5 | 65 | 65,62 | 73,30 | 46,79 | 42,16 | 54,40 | 33,93 | 8,36 | -6,58 | 2,35 | 0,83 | 6,75 | 7,21 |
| NL6 | PT6 | 85 | 70,54 | 93,51 | 75,73 | 35,84 | 44,25 | 29,24 | 7,67 | -2,23 | 4,67 | -2,98 | 7,04 | 4,78 |
| NL7 | PT7 | 60 | 67,86 | 141,15 | 79,71 | 60,27 | 65,75 | 34,53 | 7,25 | -1,12 | 2,95 | 3,37 | 4,49 | 3,00 |
| ZW1 | ACT1 | 80 | 51,83 | 120,97 | 82,66 | 65,59 | 69,21 | 61,67 | 2,73 | -4,40 | 4,98 | 5,44 | 4,36 | 2,91 |
| ZW2 | ACT2 | 55 | 97,41 | 96,01 | 84,18 | 58,78 | 52,83 | 37,08 | -1,86 | -1,59 | -4,98 | -1,13 | 6,63 | 5,28 |
| ZW4 | ACT3 | 80 | 128,05 | 145,03 | 91,75 | 97,88 | 61,74 | 54,08 | 2,71 | -8,00 | 4,39 | -0,69 | 6,98 | 4,41 |
| ZW5 | ACT4 | 65 | 72,99 | 77,85 | 46,30 | 44,04 | 76,19 | 40,42 | -2,03 | -1,42 | -1,13 | 5,56 | 4,17 | 3,99 |
| ZW6 | ACT5 | 75 | 63,33 | 77,41 | 62,84 | 66,07 | 76,32 | 50,40 | 1,84 | -2,99 | 3,30 | 3,38 | 4,27 | 2,96 |
